# Pyroglutamic acidosis as a cause for high anion gap metabolic acidosis: a prospective study

**DOI:** 10.1038/s41598-019-39257-4

**Published:** 2019-03-05

**Authors:** Shir Raibman Spector, Haim Mayan, Ronen Loebstein, Noa Markovits, Eldar Priel, Eias Massalha, Yuval Shafir, Itai Gueta

**Affiliations:** 1 Department of Medicine E, Sheba Medical Center, Tel Hashomer, Israel; 2 The institute for Clinical Pharmacology and Toxicology, Sheba Medical Center, Tel Hashomer, Israel; 3 Sourasky School of Medicine, Tel Aviv University, Tel Aviv, Israel

## Abstract

5-oxoprolinemia (pyroglutamic acid, PGA) in the absence of acetaminophen use has been rarely reported as a cause for high anion gap metabolic acidosis. We investigated the prevalence and risk factors for elevated PGA concentrations among hospitalized patients with high anion gap metabolic acidosis: We prospectively enrolled patients with high anion gap metabolic acidosis hospitalized in the department of medicine. For each patient we collected the main diagnosis, concurrent medications and laboratory parameters. Spot urine samples were tested for PGA concentration. Levels ≥63 µmol/mmol creatinine were considered elevated. Overall, forty patients were prospectively followed. Mean age was 66.9 (17.9) years. Four (6.3%) patients had a high urine PGA level and demonstrated also lower blood pH (7.2 vs 7.3, p = 0.05) and lower serum lactate concentration (17.5 mg/dl vs 23.0 mg/dl, p = 0.04). Additionally, the high PGA level group consisted of more patients with septic shock [2/4 (50%) vs 3/36 (8.3%)] with a trend towards significance (p = 0.07). In conclusion, PGA might have a role in patients with septic shock and acidosis. Being a treatable condition, PGA should be taken into consideration particularly when no other cause for high anion gap is identified.

## Introduction

High anion gap metabolic acidosis (HAGMA) is a common acid-base disturbance encountered in hospitalized patients. The most common causes are the accumulation of lactate, ketones, urea and ingestion of toxins. However, in cases where no other explanation is found, less common etiologies such as accumulation of D-lactate or pyroglutamic acid (PGA) should be suspected^1,2^. The latter has been mostly reported in the presence of chronic acetaminophen use.

To our department a 77-year-old female was admitted due to reduced level of consciousness, polyuria and fever of 38.8 °C. Her past medical history was significant for osteoporosis and a recent hip replacement with a complicated course that required its removal due to *Pseudomonas* MDR infection. She also suffered from epilepsy, ischemic heart disease, paroxysmal atrial fibrillation and had a history of ischemic stroke in recent years prior to admission. Her chronic medications included omeprazole, ipratropium bromide, risperidone, citalopram, furosemide, atorvastatin, enoxaparin, phenytoin, carbamazepine, vigabatrin and potassium supplements. Initial Laboratory evaluation showed leukocytosis with left deviation, pre-renal azotemia and a high CRP level. Her Urine dipstick was positive for leukocytes and nitrites.

A diagnosis of urinary tract infection was made and empirical treatment with *ofloxacin* was initiated. Urine cultures demonstrated *Pseudomonas aeruginosa* and based on sensitivity studies treatment was changed to *meropenem*. However, during the third day of hospitalization the patient was still somnolent and her venous blood gases demonstrated a consistent metabolic acidosis with pH 7.298, HCO3- 15.9 mmol/L, PCO2- 33.3 mmHg. The albumin adjusted anion gap was 24.5 mEq/l (normal values: 12–16 mEq/l). Blood and urine samples were negative for lactate and ketones with an osmolar gap within the normal range. Carbamazepine level was also within therapeutic range.

**Figure 1:**
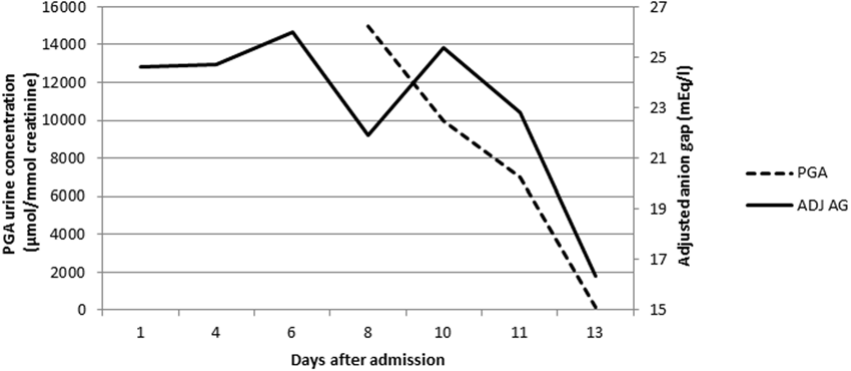
Urinary pyroglutamic acid concentrations and concomitant plasma anion gap.

Given no explanation for the high anion gap metabolic acidosis (HAGMA), vigabatrin induced pyrogluatmic acidosis was suspected. Urine sample demonstrated extremely elevated PGA levels of 15,000 µmol/mmol creatinine (normal values ≤ 63 µmol/mmol creatinine). Accordingly, vigabatrin was discontinued and N-acetylcysteine (NAC) in a dose similar to acetaminophen overdose treatment protocol was initiated. Due to slow improvement in the acidosis and the presence of PGA, two courses of hemodialysis were conducted. The patient regained consciousness and urine assay showed improving anion gap and urine PGA levels (Fig. 1).

PGA, also known as 5-oxoproline, is an intermediate metabolite in glutathione cycle. It is synthesized by γ-glutamyl cyclotransferase and catabolized by 5-oxoprolinase. The former is induced by glutathione depleted states which enhances PGA production^3,4^. Depletion is most commonly encountered secondary to acetaminophen consumption, as well as in severe sepsis, chronic alcoholism, chronic liver failure and malnutrition. Other recognized risk factors are old age, pregnancy and female gender^5,6^. On the other hand, accumulation can be secondary to inherited deficiency or the inhibition of 5-oxoprolinase^7^. Vigabatrin use has been previously reported to be associated with elevated PGA concentration^8^.

In light of the scattered reports on PGA concentration among hospitalized patients, we conducted a prospective study aiming to describe the relative contribution of PGA in patients with increased anion gap metabolic acidosis.

## Materials and Methods

### Design

A prospective study designed to examine the prevalence of elevated PGA concentrations in urine spot sample of patients with high anion gap (AG ≥ 16 mmol/l), and to identify risk factors associated with elevated levels. In order to account for drug exposure, only patients hospitalized longer than 48 hours were included. Informed consent was obtained from all study participants. All methods were carried out in accordance with relevant guidelines and regulations. The study protocol was approved by the Chaim Sheba Medical Center review board at Tel Hashomer, Israel.

### Cohort definition and data extraction

From January to December 2017 hospitalized patients were screened for high AG which was calculated using the basic formula (Na^+^ + K^+^ − Cl^−^ + HCO3^−^) and further corrected to plasma albumin levels using the Figge-Jabor-Kazda-Fencl equation (AG + 2.5x ([Normal Albumin]-[Observed Albumin])^9^. For each patient we documented demographic characteristics and the primary admission diagnosis. Sepsis was defined as confirmed infection plus exhibiting 2 of 3 general symptoms including abnormal temperature, heart rate ≥ 90 beats per minute and respiratory rate ≥ 20 per minute. Septic shock was defined as systolic pressure ≤ 90 mmHg that doesn’t adequately respond to simple fluid replacement together with severe sepsis (sepsis plus at least one major organ failure). Additional documented data included comorbidities (anemia, diabetes mellitus, hypertension, chronic renal failure, end stage renal disease, hypothyroidism, dyslipidemia, stroke, chronic heart failure, smoker, site of malignancy, previous surgeries or transplantations, cirrhosis, fatty liver, chronic obstructive pulmonary disease, asthma, thromboembolic events, G6PD deficiency, the presence and site of effusion, and obesity), as well as all medications administered within the 48 hours prior to sampling. Laboratory variables included complete blood count parameters, blood chemistry (serum albumin, sodium, chloride, potassium, phosphor, liver function tests, renal function tests, lipase and amylase, CRP, LDH, lactate, total protein and albumin) and venous blood gases. Spot urine samples were tested for ketones and PGA concentration. All laboratory tests were within 12 hours following evidence for increased anion gap.

### Pyroglutamic acid assay

Spot urine samples were tested for PGA concentration using gas chromatograph mass spectrometry. The analyses were performed on a Hewlett-Packard (PaloAlto, CA) HP5890A gas chromatograph coupled to an HP5970B mass-selective detector and an HP59940A ChemStation. Quantification of the acids was based on the specific ion masses^10^.

### Outcome definition

PGA urine concentration above the normal value was defined as higher than 63 µmol/mmol creatinine.

### Data analysis

Data are presented as mean ± standard deviation (SD) or median with interquartile range (IQR) as appropriate for continuous variables and proportions for categorical variables. Patients were divided into high PGA level group (≥63 µmol/mmol creatinine) and low PGA level group (<63 µmol/mmol creatinine). Comparison between groups was conducted using independent t-test or Mann-Whitney U test for parametric and nonparametric analysis, respectively. Categorical variables were compared using Chi-square or Fisher’s exact tests. Logistic regression analysis was employed to examine the relative association between related variables and being in the high PGA level group. All analysis were two-tailed and p-value ≤ 0.05 was considered significant. Statistical analyses were performed using SPSS software (version 21 IBM ®, SPSS ® Inc).

## Results

### Study cohort

A total of fifty patients with high anion gap metabolic acidosis were identified, of whom 10 were excluded mainly due to incomplete lab results. Thus, the final study cohort compromised 40 subjects: 19 (47.5%) females and 21 (52.5%) males with a mean age of 66.9 (17.9) years. 18 (45%) patients had chronic renal failure [mean creatinine 4.4 (2.3) mg/dl] and 19 (48%) had diabetes mellitus type II. Of the entire cohort, 20 (50.0%) were admitted due to sepsis, of whom 5 (12.5%) were diagnosed with septic shock. Mean anion gap corrected for albumin was 22.9 (3.0) mEq/l with median pH and mean bicarbonate level of 7.3 (7.2–7.4) and 19.9 (13.7) mmol/l, respectively. Median creatinine and urea were 1.7 (0.8–4.4) mg/dl and 89.0 (43.3–162.0) mg/dl, respectively. Median plasma lactate concentration was 22.5 (16.3–30) mg/dl and 7 (17.5%) patients had traces for ketones. Prior to urine sampling, paracetamol was administered to 6 (15%) patients.

### Urine pyroglutamic acid concentration

Median PGA urine concentration of the entire cohort was 28.8 (20.3–38.8) µmol/mmol creatinine. The high PGA level group (≥63 µmol/mmol creatinine) consisted of 4 (6.3%) patients of whom median PGA concentration was 72.5 (65.2–106.3) µmol/mmol creatinine.

Univariate analysis (Table 1) showed that the group with high PGA level consisted more patients with septic shock [2/4 (50%) vs 3/36 (8.3%)] with a trend towards significance (p = 0.07). This group was also characterized by lower pH (p = 0.05) and lower lactate concentration (p = 0.04).

**Table 1:** Univariate analysis of the association between the patient baseline characteristics and low and high pyroglutamic acid concentration.

	Normal range	Low PGA group	High PGA group	P value
Pyroglutamate, µmol/mmol creatinine (SD)	≤63	28.6 (12.3)	81.3 (24.2)	
N	—	36	4	
Age, years (IQR)	—	68.0 (60.0–80.3)	52.5 (44.3–84.8)	0.28
Female (%)	—	17 (47.2)	2 (50)	1.0
Diabetes mellitus type 2 (%)	—	18 (50)	1 (25)	0.61
Chronic renal failure (%)	—	16 (44.4)	2 (50)	1.0
Dialysis (%)	—	3 (8.3)	0 (0)	1.0
Acute renal failure (%)	—	5 (13.9)	0 (0)	1.0
Sepsis (%)	—	16 (44.4)	4 (100)	0.11
Shock (%)	—	3 (8.3)	2 (50)	0.07
RBC x10^6^/ml	4.2–6.1	3.9 (3.7–4.7)	3.2 (2.9–3.4)	0.16
Hb, g/dl (SD)	12–16	10.9 (2.9)	9.4 (1.3)	0.30
Creatinine mg/dl (IQR)	0.8–1.3	1.7 (0.8–4.2)	3.1 (1.0–4.5)	0.65
Urea mg/dl (IQR)	17–43	87.0 (45.3–153.6)	184.0 (63.8–199.2)	0.26
pH	7.35–7.45	7.3 (7.3–7.4)	7.2 (7.0–7.3)	**0**.**05**
Bicarbonate mmol/l (SD)	22–26	20.2 (4.3)	18.0 (4.2)	0.30
Anion Gap meq/l, Figge (SD)	12–16	22.7 (3.1)	24.3 (2.8)	0.34
Urine positive for ketones (%)	0	7 (19.4)	0 (0)	1.0
Lactate mg/dl (IQR)	4–16	23.0 (17.5–31.5)	17.5 (10.0–19.0)	**0**.**04**
Patients received paracetamol (%)	—	5 (13.9)	1 (25)	0.50

Logistic regression demonstrated only shock to be an independent risk factor associated with above normal PGA concentration (OR 33.65, p = 0.038).

## Discussion

Elevated PGA concentration is a known cause for high anion gap metabolic acidosis. However, the exact contribution to the high anion gap in hospitalized patients has been mainly described in case reports and case series mostly related to chronic acetaminophen use.

Glutathione depletion states secondary to conditions in which metabolic stress is prominent has also been associated with increased PGA levels. In our cohort, patients diagnosed with shock, a condition characterized by metabolic stress, showed higher rate of increased PGA urine level with a tendency towards significance. Noteworthy is that PGA concentrations among healthy individuals varies on a daily basis with peaks during times of metabolic stress, e.g. menstruation, and yet mostly still within normal range^11^.

Univariate analysis showed that in patients with low pH and no explanation for elevated anion gap, as demonstrated by the lack of ketones and significantly lower lactate levels, PGA urine concentrations were above normal range. The latter was further supported by the significant association between high PGA levels and shock as shown in the multivariate analysis. These observations are in line with previous reports suggesting to extend the differential diagnosis in cases where no cause of HAGMA is identified^6^. The rates of acetaminophen use did not differ between the groups of high and low PGA urine levels. This association reported in previous publications is mostly accompanied by chronic long term daily use^2,5^. However, only 6 patients in our cohort consumed acetaminophen and for a short period of time and hence was probably not powered enough to demonstrate association.

Interestingly, the patient who consumed vigabatrin for several years did not demonstrate HAGMA during hospitalizations prior to her current one. It is possible that the septic shock characterizing her described hospitalization had a major additive effect to the vigabatrin associated 5-oxoprolinemia, resulting in further glutathione depletion.

The treatment of 5-oxoprolinemia is mainly based on the etiology, PGA concentration and clinical picture. Our patient treated with vigabatrin had significant higher values as compared to the 4 patients in high PGA group. This difference is probably attributed to the additive effect of the offensive drug on the accumulation of PGA and hence such agents should be discontinued. Based on the clinical picture and laboratory values NAC treatment should be considered. Noteworthy is the high rates of anaphylactoid reactions reported to be as high as 48%^12^. However, most of these reactions are considered mild and resolves quickly after slowing down NAC infusion rate^13^. In other cases in which metabolic stress is the main etiology, treatment should address the underlying cause. Nevertheless, NAC administration in patients with septic shock is controversial with studies demonstrated variable results^14^.

Several limitations should be noted: Our cohort consisted of patients with mild to moderate acidosis reflected by their mean pH and bicarbonate values, and hence our findings might underestimate the real prevalence of elevated PGA concentrations among acidotic patients. Furthermore, since only spot urine samples were collected, we could not account for the intra-individual variability of PGA concentration. The latter is particularly emphasized given the known variation among healthy individuals; nevertheless, this variability is reported to be mostly within normal range.

In conclusion, elevated PGA concentration among hospitalized patients with unexplained HAGMA and evidence for metabolic stress should be taken in consideration. However, compared to identified offensive agents associated with 5-oxoprolinemia, PGA levels are only mildly elevated and probably do not require additional therapeutic interventions.
