# Publisher Correction: Pyroglutamic acidosis as a cause for high anion gap metabolic acidosis: a prospective study

**DOI:** 10.1038/s41598-020-70345-y

**Published:** 2020-08-04

**Authors:** Shir Raibman Spector, Haim Mayan, Ronen Loebstein, Noa Markovits, Eldar Priel, Eias Massalha, Yuval Shafir, Itai Gueta

**Affiliations:** 1 Department of Medicine E, Sheba Medical Center, Tel Hashomer, Israel; 2 The Institute for Clinical Pharmacology and Toxicology, Sheba Medical Center, Tel Hashomer, Israel; 3 Sourasky School of Medicine, Tel Aviv University, Tel Aviv, Israel

Correction to: *Scientific Reports*https://doi.org/10.1038/s41598-019-39257-4, published online 05 March 2019

In the original version of this Article, author Shir Raibman Spector was incorrectly indexed. This error has now been corrected.

